# N-terminal pro-brain natriuretic peptide – a significant biomarker of disease development and adverse prognosis in patients with exertional heat stroke

**DOI:** 10.1186/s40779-024-00531-w

**Published:** 2024-04-23

**Authors:** Long Feng, Jian-Yuan Yin, Yao-Hong Liu, Pei Zhang, Ya-Li Zhao, Qing Song, Ping Ping, Shi-Hui Fu

**Affiliations:** 1 Department of Anesthesiology, Hainan Hospital of Chinese PLA General Hospital, Sanya, 572013 Hainan China; 2 Department of Critical Care, Hainan Hospital of Chinese PLA General Hospital, Sanya, 572013 Hainan China; 3 School of Life Science, Beijing Institute of Technology, Beijing, 100081 China; 4 Central Laboratory, Hainan Hospital of Chinese PLA General Hospital, Sanya, 572013 Hainan China; 5 Heatstroke Treatment and Research Center of Chinese PLA, Hainan Hospital of Chinese PLA General Hospital, Sanya, 572013 Hainan China; 6 General Station for Drug and Instrument Supervision and Control, Joint Logistic Support Force of Chinese PLA, Beijing, 100076 China; 7 Department of Cardiology, Hainan Hospital of Chinese PLA General Hospital, Sanya, 572013 Hainan China; 8 Department of Geriatric Cardiology, Chinese PLA General Hospital, Beijing, 100853 China

**Keywords:** Exertional heat stroke, Mortality, Myocardial enzymes, N-terminal pro-brain natriuretic peptide, Physical activity

Dear Editor,

The most serious heat related injury is exertional heat stroke (EHS). EHS occurs when healthy individuals perform physical activity in a hot and humid environment [1]. A disrupted balance between heat production and dissipation in the human body results in excessive body heat storage in cases. It occurs frequently in the military population because of work characteristics such as the requirements to perform essential duties under prolonged heat stress, the need to achieve mission objectives during deployment operations, or the opportunities for training and selection for elite units [2]. The pathophysiology of EHS is complex, which often results in thermoregulation failure, hemodynamic disturbance, and endotoxin release*,* and further causes multiple organ failure, probably increasing myocardial enzymes and N-terminal pro-brain natriuretic peptide (NT-proBNP) levels. Rhabdomyolysis caused by EHS often results from mechanical and metabolic injury to the striated muscle fibers accompanied with the release of muscle contents into the circulation [3]. Liu et al. [4] also found that NT-proBNP levels were significantly higher in dead group than those in survival group in the EHS related study. There are scarce literature assessing biochemical biomarkers including myocardial enzymes and NT-proBNP in patients with EHS all around the world. Our hospital is located in Sanya, Hainan Province of China, in the tropics with long-term high temperature and humidity exposure and receives patients with EHS every year because of long exposure to field work, marathon running, and so on. The aim of the present study was to analyze whether myocardial enzymes and NT-proBNP levels were associated with the disease and the prognosis in order to provide scientific reference for identifying and managing these patients with EHS.

A total of 45 participants with EHS and 45 participants without EHS were admitted to Hainan Hospital of Chinese PLA General Hospital. All participants in the present study had ejection fraction > 50%, without reduced ejection fraction. Samples of venous blood were routinely collected by venipuncture and delivered to our biochemistry department. Red blood cell counts (RBC), white blood cell counts (WBC), and levels of albumin, total bilirubin (TB), NT-proBNP, lactate dehydrogenase (LDH), myoglobin (Mb), creatine kinase (CK), creatine kinase-MB (CK-MB), high-sensitivity cardiac troponin T (hs-cTnT), and uric acid (UA) in serum were monitored by qualified technicians without the knowledge of clinical data when the patients’ first arrival to our hospital. From June 1, 2013 to July 1, 2022, all participants were followed up for a median period of 749 (455, 1148) d.

The whole cohort had a median age of 24 (21, 30) years with males accounting for 90.0%. Participants with EHS had lower diastolic blood pressure, RBC and albumin levels, and higher WBC, TB, NT-proBNP, LDH, Mb, CK, CK-MB, hs-cTnT levels and mortality, compared with those without EHS (*P* < 0.05, Additional file 1: Table S1). Age, gender, height, systolic blood pressure and UA levels had no significant difference between participants with and without EHS (*P* > 0.05). In multivariate logistic regression analysis, NT-proBNP [Exp(*β*) = 1.069, 95%CI 1.009–1.131], LDH [Exp(*β*) = 1.027, 95%CI 1.008–1.046], Mb [Exp(*β*) = 1.029, 95%CI 1.007–1.052], CK [Exp(*β*) = 1.005, 95%CI 1.002–1.008] and CK-MB [Exp(*β*) = 1.038, 95%CI 1.004–1.074] levels were significantly and independently associated with EHS (*P* < 0.05). However, the associations of hs-cTnT and UA levels with EHS did not reach statistical significance (*P* > 0.05, Additional file 1: Table S2). In multivariate Cox regression analysis, NT-proBNP level [Exp(*β*) = 1.002, 95%CI 1.000–1.004] was significantly and independently associated with mortality (*P* < 0.05), while LDH, Mb, CK, CK-MB, hs-cTnT and UA levels had no significant associations with mortality (*P* > 0.05, Additional file 1: Table S3).

The present study indicated that NT-proBNP level was significantly associated with the occurrence and prognosis of EHS. NT-proBNP levels are low at rest in professional athletes and the increase after physical exercise may be physiological [5]. An increase in NT-proBNP levels is thought to be associated with vigorous physical exercise and heavy cardiac effort [5]. In the hot and humid environment, EHS is often caused by prolonged or heavy physical activity, and NT-proBNP levels increase in healthy athletes after prolonged or strenuous exercise. Myocardial hypertrophy and dysfunction are reported in patients with EHS, and NT-proBNP may be used as a significant biomarker of EHS. NT-proBNP levels have also been associated with an increased mortality in population-based studies with long-term follow-up, suggesting NT-proBNP as a predictor of mortality in the general population. This is also true for younger and healthier individuals without myocardial hypertrophy and dysfunction. The present study realized that NT-proBNP not only has a significant relationship with EHS, but also provides prognostic information for EHS. Clinical prognosis is the best indicator of disease severity, and NT-proBNP levels were correlated with the severity of EHS.

Patients who develop EHS have myocardial injury and traction without reaching the stage of myocardial infarction, and myocardial injury resulting from EHS is modifiable rather than leading to severe damage or even death [2]. It was found that physical exercise could affect NT-proBNP levels, but no severe myocardial injury was found in healthy individuals. After physical exercise, NT-proBNP elevation is not caused by myocardial injury but has cytoprotective effects, whereas the release of myocardial enzymes should be considered irreversible injury or myocardial infarction [4]. The present study illustrated that NT-proBNP rather than myocardial enzymes was significantly associated with adverse prognosis in patients with EHS, suggesting that NT-proBNP is superior to myocardial enzymes as an important biomarker of EHS to evaluate the occurrence and prognosis of EHS [5].

The present study had the following limitations. Firstly, this study about EHS was performed in the tropics (Sanya) and may have regional characteristics and differences. EHS is more prevalent in the tropics and is a disease with regional characteristics, and our location provides the required and advantaged condition for the present study about EHS. Regional characteristics are derived from EHS itself rather than our study. Secondly, this study was a single-center clinical study with small number of patients. Because the prevalence of EHS is small, the number of patients with EHS is relatively large in Sanya of tropics compared with other regions. The present study remains to be confirmed by multicenter studies and functional experiments.

In conclusion, EHS mainly occurs during a long period of physical activity in the hot and humid environment, causing high mortality in the world and requiring medical attention and emergent treatment. The present study demonstrated that NT-proBNP level was not only clearly associated with the occurrence of EHS, but also significantly associated with an increased mortality in such patients. This suggests that NT-proBNP may serve as a significant biomarker of disease development and adverse prognosis in patients with EHS.

### Supplementary Information

**Additional file 1: Table S1** Characteristics of participants with and without EHS. **Table S2 **Biomarkers associated with EHS in multivariate logistic regression analysis. **Table S3 **Biomarkers associated with prognosis in multivariate Cox regression analysis.

## Data Availability

All data and materials are available under the requirement to the corresponding authors.

## Abbreviations

CK: Creatine kinase
CK-MB: Creatine kinase-MB
EHS: Exertional heat stroke
hs-cTnT: High-sensitivity cardiac troponin T
LDH: Lactate dehydrogenase
Mb: Myoglobin
NT-proBNP: N-terminal pro-brain natriuretic peptide
RBC: Red blood cell counts
UA: Uric acid
WBC: White blood cell counts
