# Supplementary material for: N-terminal pro-brain natriuretic peptide – a significant biomarker of disease development and adverse prognosis in patients with exertional heat stroke
Source: Mil Med Res. 2024 Apr 23;11:26. doi: 10.1186/s40779-024-00531-w (PMC11036771; doi:10.1186/s40779-024-00531-w)
Supplement: Supplementary file 1 — Additional file 1: Table S1 Characteristics of participants with and without EHS. Table S2 Biomarkers associated with EHS in multivariate logistic regression analysis. Table S3 Biomarkers associated with prognosis in multivariate Cox regression analysis. [file 40779_2024_531_MOESM1_ESM.pdf]

Table S1 Characteristics of participants with and without EHS

**Characteristics**	**Total (*n* = 90)**	**Without EHS (*n* = 45)**	**With EHS (*n* = 45)**	***P*-value**
Age [year, $M(Q_1, Q_3)$]	24 (21, 30)	24 (21, 30)	23 (21, 32)	0.945
Gender [*n*(%)]				0.292
Male	81 (90.0)	39 (86.7)	42 (93.3)	
Female	9 (10.0)	6 (13.3)	3 (6.7)	
Height [cm, $M(Q_1, Q_3)$]	172.0 (168.0, 177.0)	172.0 (168.0, 177.5)	172.0 (167.5, 177.0)	0.942
SBP [mmHg, $M(Q_1, Q_3)$]	120.0 (112.8, 127.0)	121.0 (116.5, 126.5)	118.0 (107.5, 127.5)	0.202
DBP [mmHg, $M(Q_1, Q_3)$]	72.0 (66.0, 78.0)	76.0 (70.0, 84.0)	68.0 (58.0, 76.0)	< 0.001
RBC [$\times 10^{12}$/L, $M(Q_1, Q_3)$]	4.75 (4.21, 5.14)	5.11 (4.72, 5.34)	4.35 (3.99, 4.76)	< 0.001
WBC [$\times 10^{9}$/L, $M(Q_1, Q_3)$]	7.34 (5.88, 9.24)	6.73 (5.58, 8.08)	7.94 (6.10, 11.60)	0.012
Albumin [g/L, $M(Q_1, Q_3)$]	40.95 (36.63, 44.95)	43.80 (40.95, 45.90)	37.10 (33.80, 40.95)	< 0.001
TB [μmol/L, $M(Q_1, Q_3)$]	13.40 (8.78, 20.35)	11.00 (8.60, 16.70)	16.55 (9.25, 24.55)	0.033
Biomarkers				
NT-proBNP [pg/ml, $M(Q_1, Q_3)$]	141.6 (24.8, 311.5)	26.0 (15.0, 51.5)	309.0 (188.0, 614.0)	< 0.001
LDH [U/L, $M(Q_1, Q_3)$]	179.0 (149.8, 253.3)	154.0 (137.0,169.0)	241.0 (193.5, 540.5)	< 0.001
Mb [ng/ml, $M(Q_1, Q_3)$]	37.9 (21.0, 142.0)	23.0 (21.0, 34.4)	127.0 (41.0, 2115.0)	< 0.001
CK [U/L, $M(Q_1, Q_3)$]	256.00 (116.25, 1504.75)	119.00 (79.50, 165.00)	1404.00 (454.50, 2988.50)	< 0.001
CK-MB [U/L, $M(Q_1, Q_3)$]	15.40 (11.10, 37.53)	12.10 (9.80, 14.35)	35.40 (18.95, 62.90)	< 0.001
hs-cTnT [pg/ml, $M(Q_1, Q_3)$]	7.0 (5.0, 27.0)	5.0 (4.0, 7.0)	14.0 (7.0, 143.0)	< 0.001
UA [μmol/L, $M(Q_1, Q_3)$]	354.0 (305.8, 445.8)	367.0 (314.5, 426.0)	331.0 (265.5, 501.5)	0.526
Mortality [*n*(%)]	5 (5.6)	0	5 (11.1)	0.021

EHS exertional heat stroke, *SBP* systolic blood pressure, *DBP* diastolic blood pressure, *RBC* red blood cell counts, *WBC* white blood cell counts, *TB* total bilirubin, *NT-proBNP* N-terminal pro-brain natriuretic peptide, *LDH* lactate dehydrogenase, *Mb* myoglobin, *CK* creatine kinase, *CK-MB* creatine kinase-MB, *hs-cTnT* high-sensitivity cardiac troponin T, *UA* uric acid

Table S2 Biomarkers associated with EHS in multivariate logistic regression analysis

**Biomarkers**	**Exp(*β*)**	**95%CI**	***P*-value**
NT-proBNP	1.069	1.009 – 1.131	0.023
LDH	1.027	1.008 – 1.046	0.006
Mb	1.029	1.007 – 1.052	0.010
CK	1.005	1.002 – 1.008	0.004
CK-MB	1.038	1.004 – 1.074	0.029
hs-cTnT	1.075	0.997 – 1.160	0.061
UA	1.002	0.996 – 1.008	0.523

EHS exertional heat stroke, *NT-proBNP* N-terminal pro-brain natriuretic peptide, *LDH* lactate dehydrogenase, *Mb* myoglobin, *CK* creatine kinase, *CK-MB* creatine kinase-MB, *hs-cTnT* high-sensitivity cardiac troponin T, *UA* uric acid

Table S3 Biomarkers associated with prognosis in multivariate Cox regression analysis

**Biomarkers**	**Exp(*β*)**	**95%CI**	***P*-value**
NT-proBNP	1.002	1.000 – 1.004	0.044
LDH	1.001	1.000 – 1.002	0.088
Mb	1.000	1.000 – 1.000	0.566
CK	1.000	1.000 – 1.000	0.701
CK-MB	1.001	0.991 – 1.010	0.920
hs-cTnT	1.000	0.999 – 1.001	0.899
UA	1.002	0.995 – 1.010	0.565

NT-proBNP N-terminal pro-brain natriuretic peptide, *LDH* lactate dehydrogenase, *Mb* myoglobin, *CK* creatine kinase, *CK-MB* creatine kinase-MB, *hs-cTnT* high-sensitivity cardiac troponin T, *UA* uric acid
